# Use of a Self-guided Computerized Cognitive Behavioral Tool During COVID-19: Evaluation Study

**DOI:** 10.2196/26989

**Published:** 2021-05-31

**Authors:** Isadora Detweiler Guarino, Devin R Cowan, Abigail M Fellows, Jay C Buckey

**Affiliations:** 1 Space Medicine Innovations Laboratory Geisel School of Medicine at Dartmouth Lebanon, NH United States

**Keywords:** computerized cognitive behavioral therapy, interactive media, COVID-19, computer-based therapy, usability, acceptability, cognitive behavioral therapy, therapy, effectiveness, digital health, depression, stress

## Abstract

**Background:**

Internet-based programs can help provide accessible and inexpensive behavioral health care to those in need; however, the evaluation of these interventions has been mostly limited to controlled trials. Data regarding patterns of use and effectiveness of self-referred, open-access online interventions are lacking. We evaluated an online-based treatment designed to address stress, depression, and conflict management, the Dartmouth PATH Program, in a freely available and self-guided format during the COVID-19 pandemic.

**Objective:**

The primary aim is to determine users’ levels of stress and depression, and the nature of problems and triggers they reported during the COVID-19 pandemic. A secondary objective is to assess the acceptability and usability of the PATH content and determine whether such a program would be useful as a stand-alone open-access resource. The final objective is understanding the high dropout rates associated with online behavioral programs by contrasting the use pattern and program efficacy of individuals who completed session one and did not return to the program with those who came back to complete more sessions.

**Methods:**

Cumulative anonymous data from 562 individuals were analyzed. Stress triggers, stress responses, and reported problems were analyzed using qualitative analysis techniques. Scores on usability and acceptability questionnaires were evaluated using the sign test and Wilcoxon signed rank test. Mixed-effects linear modeling was used to evaluate changes in stress and depression over time.

**Results:**

A total of 2484 users registered from April through October 2020, most of whom created an account without initiating a module. A total of 562 individuals started the program and were considered in the data analysis. The most common stress triggers individuals reported involved either conflicts with family or spouses and work or workload. The most common problems addressed in the mood module were worry, anxiousness, or stress and difficulty concentrating or procrastination. The attrition rate was high with 13% (21/156) completing the conflict module, 17% (50/289) completing session one of the mood module, and 14% (16/117) completing session one of the stress module. Usability and acceptability scores for the mood and stress modules were significantly better than average. In those who returned to complete sessions, symptoms of stress showed a significant improvement over time (*P*=.03), and there was a significant decrease in depressive symptoms over all time points (*P*=.01). Depression severity decreased on average by 20% (SD 35.2%; *P*=.60) between sessions one and two.

**Conclusions:**

Conflicts with others, worry, and difficulty concentrating were some of the most common problems people used the programs to address. Individuals who completed the modules indicated improvements in self-reported stress and depression symptoms. Users also found the modules to be effective and rated the program highly for usability and acceptability. Nevertheless, the attrition rate was very high, as has been found with other freely available online-based interventions.

**Trial Registration:**

ClinicalTrials.gov NCT02726061; https://clinicaltrials.gov/ct2/show/NCT02726061

## Introduction

Community epidemiological surveys estimate that as many as 30% of adults in the United States are affected by a mental disorder, yet less than half see a physician, and only a quarter are treated properly [1,2]. A number of barriers limit access to cognitive behavioral therapy (CBT). It is often not widely available, in part due to a lack of adequately trained CBT professionals [3], high cost, potential stigma, inconvenient hours, demands of attending in-person treatments, and concerns over privacy [4].

Technology can increase access to care by providing secure, inexpensive, and easily accessible treatment tools. Internet-delivered CBT (ICBT) has existed for 20 years, and a number of controlled trials and meta-analytic reviews have demonstrated the effectiveness of this approach [5-10]. Although studies have shown ICBT can be as effective as conventional face-to-face therapy [7,8], dropout rates remain exceptionally high. For example, a study has found that as few as 1% of total users completed a full course of an open-access nontracked online program, and fewer than 25% of participants completed programs in a research trial setting [11]. Meta-analytic studies have shown that self-guided web-based interventions (defined as interventions that patients work through on their own without support or guidance) exhibit less promising results than guided web-based interventions (defined as interventions that are delivered with support from a therapist or coach) [6,12-16]. However, the evaluation of web-based programs has been mostly limited to controlled trials, rather than open-access interventions [17], restricting the interpretations of the efficacy and feasibility of these programs in an unstructured format.

Our study evaluated a web-based program in a freely available format. The Dartmouth PATH Program is a multimedia-based computerized CBT tool designed to address stress, depression, and conflict management. The program was developed as a psychosocial training and treatment resource for the National Aeronautics and Space Administration (NASA) with the aim of addressing psychological challenges endured by astronauts on long duration spaceflights [18]. The operational demands of living in such isolated, confined environments can induce conflict, stress, and depression [19,20]. The PATH program has already been tested in extreme environments, such as the Hawaii Space Exploration Analog and Simulation (HI-SEAS) Mars analog and Australian Antarctic stations, and was shown to be acceptable, usable, and valuable [18,21].

Comparably, the COVID-19 pandemic has also been associated with mental health challenges related directly to the virus’ morbidity and mortality, and indirectly by the impact of physical distancing and stay-at-home orders [22]. According to the Centers for Disease Control and Prevention, symptoms of anxiety and depressive disorders increased considerably in the United States during the period of April through October 2020 compared to the same period in 2019 [23,24]. Although the PATH program was made available to the public initially in June 2016, it grew in popularity during the pandemic through news outlets and social media coverage, which increased website traffic. This popularity was largely due to the psychological challenges that were present during that time, leading to an increase in interest of news stories focusing on how people could address their psychological problems on their own. This provided a unique opportunity to determine the type of problems driving people to self-help tools and to assess the uptake, completion, and effectiveness of this ICBT resource in a self-referred, open-access fashion in comparison to its previous evaluations in controlled clinical trials.

The program was freely available as part of NCT02726061. Participants needed to agree to participate using an online consent form. We evaluated responses to the PATH program during the COVID-19 pandemic from April through October 2020. The data collected were fully anonymous and self-reported. Our objectives were to determine the levels of stress and depression as well as the nature of problems affecting individuals during the pandemic, assess the acceptability and usability of the PATH content and determine whether such a program would be useful as a stand-alone open-access resource, and understand the high dropout rates associated with online behavioral programs by contrasting the use pattern and program efficacy of individuals who completed session one and did not return to the program with those who came back to complete more sessions.

## Methods

### The PATH Program

The PATH program is an interactive, media-intensive CBT-based program that interacts with users in real time and delivers individualized feedback based on self-reported responses (Multimedia Appendix 1). In addition to self-assessment questionnaires and manuals, the program contains three primary modules: conflict management, stress management, and depression treatment. The user can complete the program by following each module’s guided instructions session by session in sequential order. Alternatively, the participant can use the self-assessments, which consist of questionnaires that identify the main problems affecting participants and redirects them to the appropriate content module.

### Conflict Module

The conflict module teaches participants how to approach conflict and reach effective solutions using CBT principles. It includes a conflict briefing, an interactive conflict simulation, a cognitive restructuring exercise, and a training module on interest-based negotiation [18]. An evaluation study in the isolated and confined HI-SEAS III expedition found this module to be useful, valuable, and interesting [18].

At the end of each one of the four sections in the conflict module, users received a survey on how valuable, feasible, and realistic the simulation was on a scale of 0 to 4 ranging from “Strongly disagree” to “Strongly agree.” The questions covered whether the activity had too much or too little information and if it was interesting or valuable.

### Stress Module

Like the conflict module, the stress module also uses CBT principles and focuses on stress management and resilience training. The module consists of six approximately 1-hour sessions. A randomized controlled trial of a version of the stress module reported significant reductions in perceived stress and increases in perceived control over stress [25]. Each session focuses on teaching participants different methods on how to deal with thoughts, feelings, and actions associated with stress. The sessions contained a mixture of activities from three major domains of feelings, thoughts, and actions. “Feelings” activities included guided muscle relaxation and focused breathing. “Thoughts” activities included compartmentalization and weighing evidence, aiming to educate the user on cognitive flexibility. Compartmentalization required the user to imagine a stressful scenario and proceed to shift their attention to perform a task quickly and accurately without being distracted by the previous stressful image. Weighing evidence used cognitive restructuring to help participants identify and dispute the validity of an automatic negative thought by weighing the evidence for and against that thought with the goal of reaching a rational conclusion. Finally, “Action” activities included effective communication, strategic problem solving, and resilience through writing. Effective communication taught assertive communication strategies, strategic problem solving involved problem-solving therapy, and resilience through writing consisted of a journaling activity [25]. At the end of each session, participants received a printout summary that included a stress profile based on the selected stressful triggers, thoughts, physical feelings, emotional feelings, current actions, and selected resilience strategies. It also contained a strategic problem-solving action plan to address the selected stressful trigger and a resilience practice plan consisting of exercises learned in that session to be practiced in the upcoming week.

Users’ progress was tracked at the beginning of each session through a self-reported survey regarding satisfaction with their progress since the last session on a binary scale (“It went well,” “It went not so well”). Participants were also asked how often they practiced each skill learned in the previous session on a scale ranging from 0 to 3 (0 “none,” 1 “once or twice,” 2 “every other day,” and 3 “daily”).

Participants completed the Perceived Stress Scale-14 (PSS) questionnaire at the beginning of each session to assess the degree to which events were perceived as stressful since their last visit. The questions in the PSS were meant to convey feelings and thoughts experienced in the last month on a 0 to 4 scale ranging from “never” to “very often.” The questionnaire contains 14 questions, and the final score is obtained by reversing the scores on seven of the positively stated items and then summing across all 14 questions. The PSS has established adequate internal and test-retest reliability, and correlates with life event scores [26].

### Depression Module

The depression module uses problem-solving treatment and consists of six sessions lasting 30 to 60 minutes. In these sessions, a mentor guides users through a step-by-step problem-solving therapy tool. Participants were first asked to identify and clarify a problem, establish an achievable goal, brainstorm solutions to the problem, evaluate pros and cons of each solution, develop an action plan to implement the selected solutions, and finally schedule enjoyable activities they will do during the next week. At the end of each session, a summary printout was available containing the problem selected, the action plan developed in that session, and a list of the selected scheduled enjoyable activities to be completed before the following session [27]. The program provides tailored feedback through branching algorithms based on user choices in their problem-solving efforts and their scores on the depression questionnaire. A randomized clinical trial on an earlier version of the module showed significant improvements in depression outcomes when using this program compared to a no treatment control group [28].

Participant progress was tracked through surveys regarding satisfaction with the amount of effort spent trying to solve the problem on a 1 to 10 scale ranging from “not satisfied at all” to “extremely satisfied” at the beginning of each session.

Users completed the Patient Health Questionnaire-9 (PHQ-9) Item Depression Scale at the beginning of each session to assess depression symptoms they had experienced for the past 2 weeks. The questionnaire had participants rating nine questions concerning their depression symptoms on a 0 to 3 scale ranging from “not at all” to “nearly every day.” The final score was calculated by adding up the total scores from each question, which ranges from 0 to 27 (0-4: no depression; 5-9: mild depression; 10-14: moderate depression; 15-19: moderate to severe depression; ≥20: severe depression). The questionnaire has demonstrated strong test-retest reliability and internal consistency [29].

### Participants

The study was approved by the Dartmouth Committee for the Protection of Human Subjects. The online program was made freely available to anyone interested in participating in the study. Users were required to accept an online consent form and then create a username and password of their choice. The data were acquired during the period of the COVID-19 pandemic from April through October 2020.

### Procedures

Users were able to browse the program’s website freely and to choose to go through the cognitive behavioral modules they selected at their own pace. The main page contains all the potential choices for users to choose from, which includes the three primary modules of depression, stress, and conflict management; a guide on how to use the program; self-assessments to guide users on finding relevant content based on individual needs; and other resources and publications.

### Demographic Questionnaire

Participant age and gender were collected upon signing up in the study.

### Usability and Acceptability Measures

The Post-Study System Usability Questionnaire (PSSUQ) is a 19-item self-report questionnaire used to assess user satisfaction with system usability at the end of a study.

The items are scored on a 7-point scale (1-7) on the strength of agreement with each statement (eg, “It was simple to use this system.” “The interface of this system was pleasant.”). The scale ranges from “Strongly agree” (1) to “Strongly disagree” (7), and a “Not applicable” indicates answers outside the scale. The overall score is obtained by summing across scores for all questions (1-19), and the evaluation is further subdivided into three subscales: system usefulness (questions 1-8), information quality (questions 9-15), and interface quality (questions 16-18). The overall scale and its subscales have shown adequate levels of reliability, validity, and sensitivity [30,31]. Initially, the program was programmed to present the PSSUQ after the third session in the stress or mood modules. When it became apparent that few people were returning for three sessions, the usability questionnaire was moved to after the first, fourth, and sixth sessions. This meant PSSUQ data were not collected for participants who visited before this change was made.

The Acceptability of Self-Guided Treatment (AST) is a 16-item self-report questionnaire developed in previous research on an earlier version of the depression module as a stand-alone treatment for depression [32]. It comprises of 16 statements (eg, “Computer programs can help with emotional problems such as depression.” “I would feel comfortable using this program without a clinician’s supervision.”) scored on a 7-point scale (1-7) ranging from “Strongly disagree” (1) to “Strongly agree” (7). This question was presented after the first, fourth, and sixth sessions in the mood and stress modules.

### Statistical Analysis

To evaluate for statistically significant improvements in PSS (stress module) and PHQ-9 (mood module) scores, we performed the nonparametric Wilcoxon signed rank test using MATLAB (MathWorks). Since the dropout rate of sessions three to six were too high, this test was only performed to assess the median of differences of paired samples for both PSS and PHQ-9 questionnaires from sessions one and two at a 5% significance level (MATLAB’s default value). The PSSUQ and AST analyses were also performed using the sign test to evaluate whether the given scores were statistically significant against the neutral mean value of each questionnaire’s scale. A right-sided test for the median of the AST scores and a left-sided test for the median of the PSSUQ scores were performed at a 5% significance level. This tested the hypothesis that the median scores of the AST questionnaires were higher than neutral (more acceptable) and that the median scores of the PSSUQ questionnaire were lower than neutral (more usable). To determine whether stress and depression would show statistically significant improvement over time, we conducted linear mixed-effects modeling to account for the time-varying natures of the variables and missing data. We entered time as the fixed effect and the intercept of subjects as random effects into the model. ATLAS.ti software (ATLAS.ti Scientific Software Development GmbH) was used to synthesize the qualitative data for both the stress and mood modules (stress triggers, emotional response to perceived stressors, and problems selected in the mood module). The numerical values are presented in this study with mean (SD), median (range), or both. The statistical analyses were conducted using MATLAB *R2020a (v9.8.0).*

## Results

### Demographics and Dropout Rate

Between April and October 2020, a total of 2484 users registered with the program website in response to media coverage of the program during the COVID-19 pandemic. Of those who registered, 1321 (53.2%) self-identified as females and 1042 (41.9%) as males with a mean age for the entire group of 44 (SD 15.2) years. The majority of those who registered did not interact with the program and therefore were not included in the treatment dropout rate. The dropout rate involves leaving treatment before its completion [33] and may occur at any point throughout the treatment. For example, a user may withdraw from the program before interacting with any of the module sessions (pretreatment dropout), prior to completion of treatment sessions at any point once treatment had started (treatment dropout), or prior to completing follow-up assessments (follow-up dropout).The majority of registered users created an account but did not proceed with any of the program sessions (pretreatment dropout) [34].

**Figure 1:**
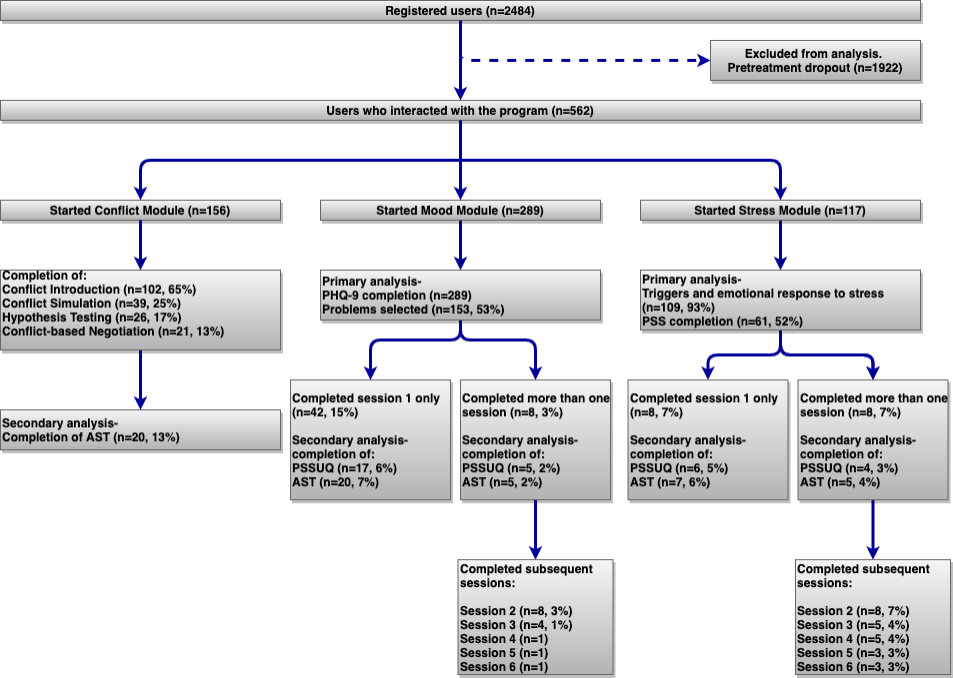
Flow of participants through a self-guided behavior therapy for conflict, mood, and stress. AST: Acceptability of Self-Guided Treatment; PHQ-9: Patient Health Questionaire-9; PSS: Perceived Stress Scale-14; PSSUQ: Post-Study System Usability Questionnaire.

A total of 562 participants interacted with the program modules and were included in the data analysis. At baseline, 156 users interacted with the conflict module, 289 with the mood module, and 117 with the stress module. The conflict module had four subsections and was completed by 21 users (Figure 1). Session one of the mood module was completed by 50 individuals, 8 of which went on to complete subsequent sessions. The PSSUQ and AST questionnaires following session one had a lower completion rate with 22 individuals completing the PSSUQ and 25 completing the AST questionnaire. Subsequent sessions had a very high attrition rate, where 8 individuals completed session two, 4 completed session three, and 1 individual completed sessions four to six. The stress module was completed by 16 individuals, 8 of which went on to complete subsequent sessions within the module. The PSSUQ and AST questionnaires were completed by 10 and 12 users, respectively. Subsequent sessions also had a high attrition rate, where 8 participants completed session two, 5 completed session three and four, and 3 users completed sessions five and six. The primary analysis included the nature of problems and stress triggers experienced by all participants. The secondary analysis focused on the usability and acceptability differences perceived by those who completed session one and did not return to the program with those who came back to complete more sessions to evaluate potential causes for treatment dropout rates.

### Conflict Module

The Conflict Introduction Questionnaire was completed by 102 participants (Table 1) and was found to be interesting (mean 3.2). The Conflict Simulation Questionnaire indicated that participants (n=39) felt like the simulation was valuable (mean 3.2). From the Hypothesis Testing Questionnaire, participants (n=26) indicated that the program was somewhat easy to understand (mean 2.5), and the feedback somewhat helped them understand why responses were correct or incorrect (mean 2.5). Participants (n=21) found the conflict-based negotiation activity enjoyable (mean 3.1) and highly valuable for learning about conflict management (mean 3.3).

**Table 1:** The evaluation of conflict module scored on a 0-4 scale ranging from “strongly disagree” (0) to “strongly agree” (4).^a^

Item		Mean (SD)	*P* value	Median (range)
**Conflict introduction (n=102)**				
	The conflict management briefing contained too little information.	1.2 (1.1)	<.001	1.0 (0-3)
	The conflict management briefing contained too much information.	0.9 (0.9)	<.001	1.0 (0-4)
	I learned a lot from the briefing.	2.8 (1.0)	<.001	3.0 (0-4)
	The conflict management briefing was interesting.	3.2 (1.0)	<.001	3.0 (0-4)
	I learned a lot from the conflict management briefing that I will probably use in future conflicts.	2.9 (1.0)	<.001	3.0 (0-4)
**Conflict simulation (n=39)**				
	Dr Greenhalgh gave too much information in his spoken comments and advice during the simulation.	0.9 (0.9)	<.001	1.0 (0-3)
	Dr Greenhalgh gave too little information in his spoken comments and advice during the simulation.	1.0 (1.0)	<.001	1.0 (0-3)
	Overall, I found the simulation valuable for learning about conflict management.	3.2 (0.8)	<.001	3.0 (1-4)
**Hypothesis testing (n=26)**				
	The process of hypothesis testing was easy to understand.	2.5 (1.1)	.048	2.5 (0-4)
	The reasons for doing hypothesis testing was easy to understand.	2.9 (1.2)	<.001	3.0 (0-4)
	The feedback on my choices helped me to understand why responses were correct or incorrect.	2.5 (1.3)	.06	3.0 (0-4)
	I was confused about what I was supposed to do in the hypothesis testing activity.	1.5 (1.1)	.03	1.5 (0-4)
**Conflict-based negotiation (n=21)**				
	Mr Weiss gave too much information in his spoken comments and advice during the interest-based negotiation activity.	1.2 (1.1)	0.2	1.0 (0-3)
	Overall, I found doing the negotiation activity enjoyable.	3.1 (0.9)	<.001	3.0 (1-4)
	Overall, I found the activity valuable for learning about conflict management.	3.3 (0.7)	<.001	3.0 (2-4)

^a^Steve and John were two characters in the simulated conflict. A sign test was performed to evaluate whether the median acceptability scores were significantly different than a median of 2 (neutral).

### Stress Module

#### Effects on Stress

**Figure 2:**
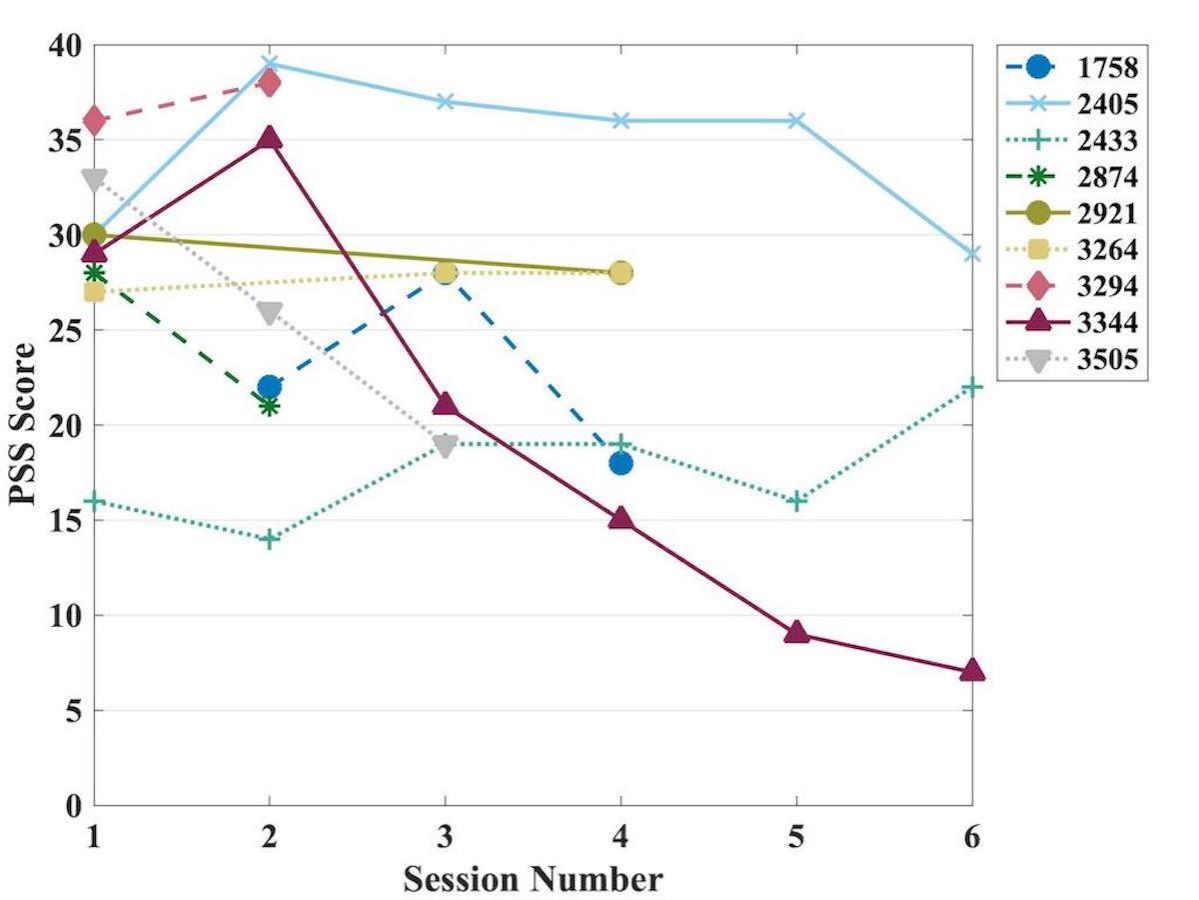
Change in stress levels across sessions one through six measured using PSS scores. The figure legend's four-digit values correspond to the identification numbers randomly assigned to users during program registration. PSS: Perceived Stress Scale-14.

The mean PSS score baseline for session one, including all participants (n=61), was 30.0 (SD 6.6). The average PSS of session one for those who returned for more than one session (n=8) was 28.6 (SD 5.9) while session two (n=8) was 27.3 (SD 9.1). The difference in scores for each participant between the first and last completed sessions was analyzed using the Wilcoxon signed rank test and was found to not be significant (*P=*.19). The individual analysis of the PSS scores across sessions one through six is shown in Figure 2.

To determine whether stress symptoms would improve significantly over time, we used a linear mixed-effects analysis of the relationship between time and PSS scores for each participant throughout all sessions completed. We entered time as the fixed effect and the intercept of subjects as random effects into the model. The decrease in symptoms in relation to time was significant (β=–.14, SE 0.062, 95% CI –0.27 to –0.013; *P*=.03).

#### Stress Troubleshooting Questionnaire

At the beginning of sessions two, four, and six, participants were asked a follow-up question to evaluate how successful participants were in achieving their goals. The question “How did it go solving this problem?” used a binary scale consisting of the responses “It went well” and “It went not so well.” A total of 16 individuals responded to this question in session two, of which 73% had a positive response. A total of 5 individuals responded to this question in session four, with an 80% positive response. Lastly, session six had a total of 3 participants answering with 100% positive responses.

#### Triggers and Emotional Response to Stress

The stress triggers and the emotional response to perceived stressors were compared for those who completed one (n=102) versus those who completed multiple sessions (n=7). Individuals were able to select multiple responses (ie, multiple triggers and emotional responses).

The stress triggers were grouped into 10 major categories: (1) conflicts with family or spouse; (2) work or workload; (3) conflicts with friends or neighbors; (4) financial concerns; (5) health concerns; (6) boredom and lack of productivity; (7) time away from friends and family; (8) internal stressors, which included negative views about oneself, uncertainty of future, and fear of failure; (9) COVID-19 and isolation; and (10) current political climate. The responses were categorized into each of the major groups, and only one category was counted per person (eg, if a participant noted multiple triggers of the same category, the analysis considered it as just one instance).

**Figure 3:**
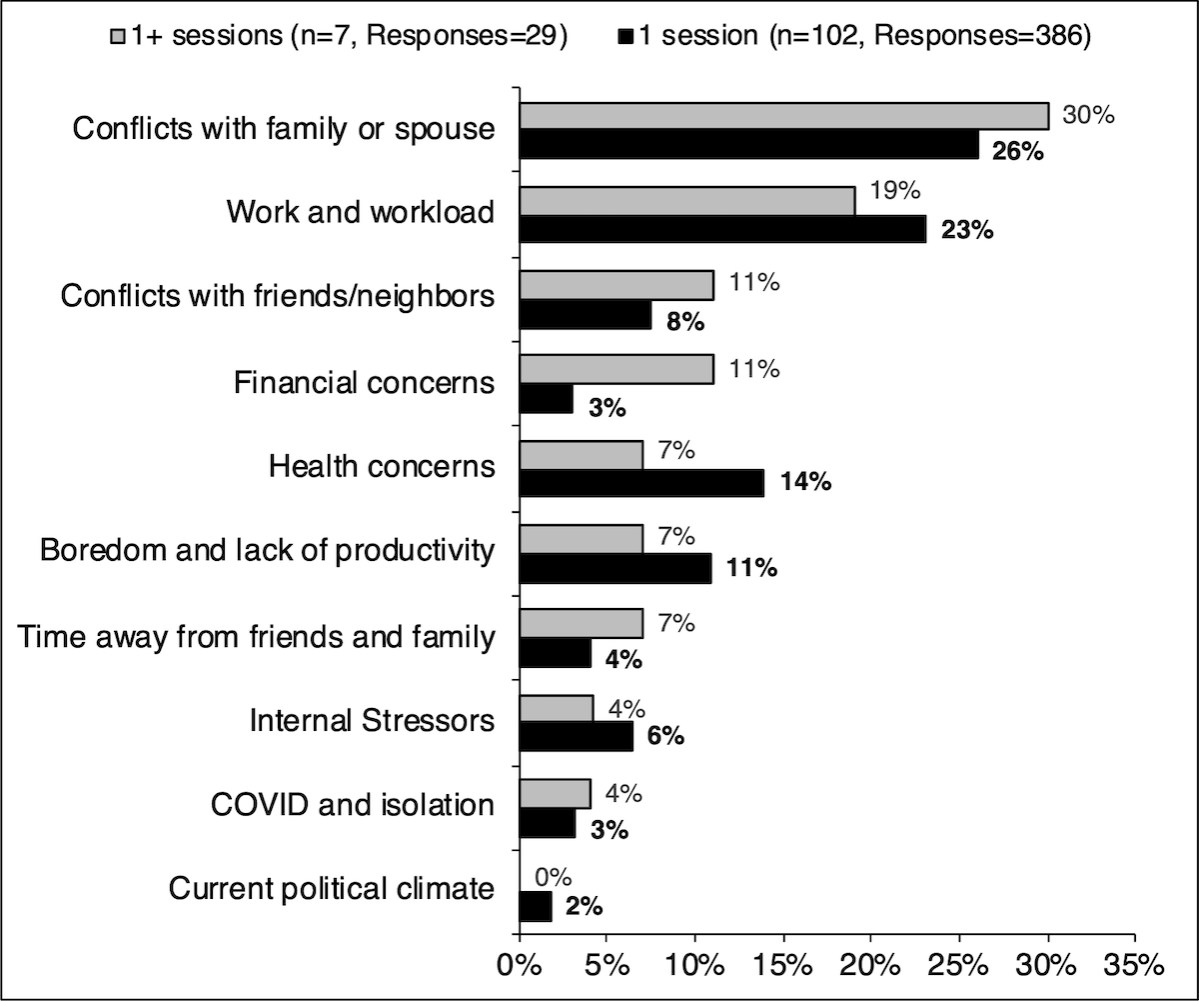
Most common stressors experienced by those who completed one versus more than one session.

Conflict with family or spouse and work or workload were the two most common stressful triggers among participants. In contrast, conflicts with friends or neighbors and financial concerns were ranked third by those who completed multiple sessions, while health concerns and boredom or lack of productivity were ranked third by those who completed just one session. The response percentage rate for each category can be found in Figure 3.

**Figure 4:**
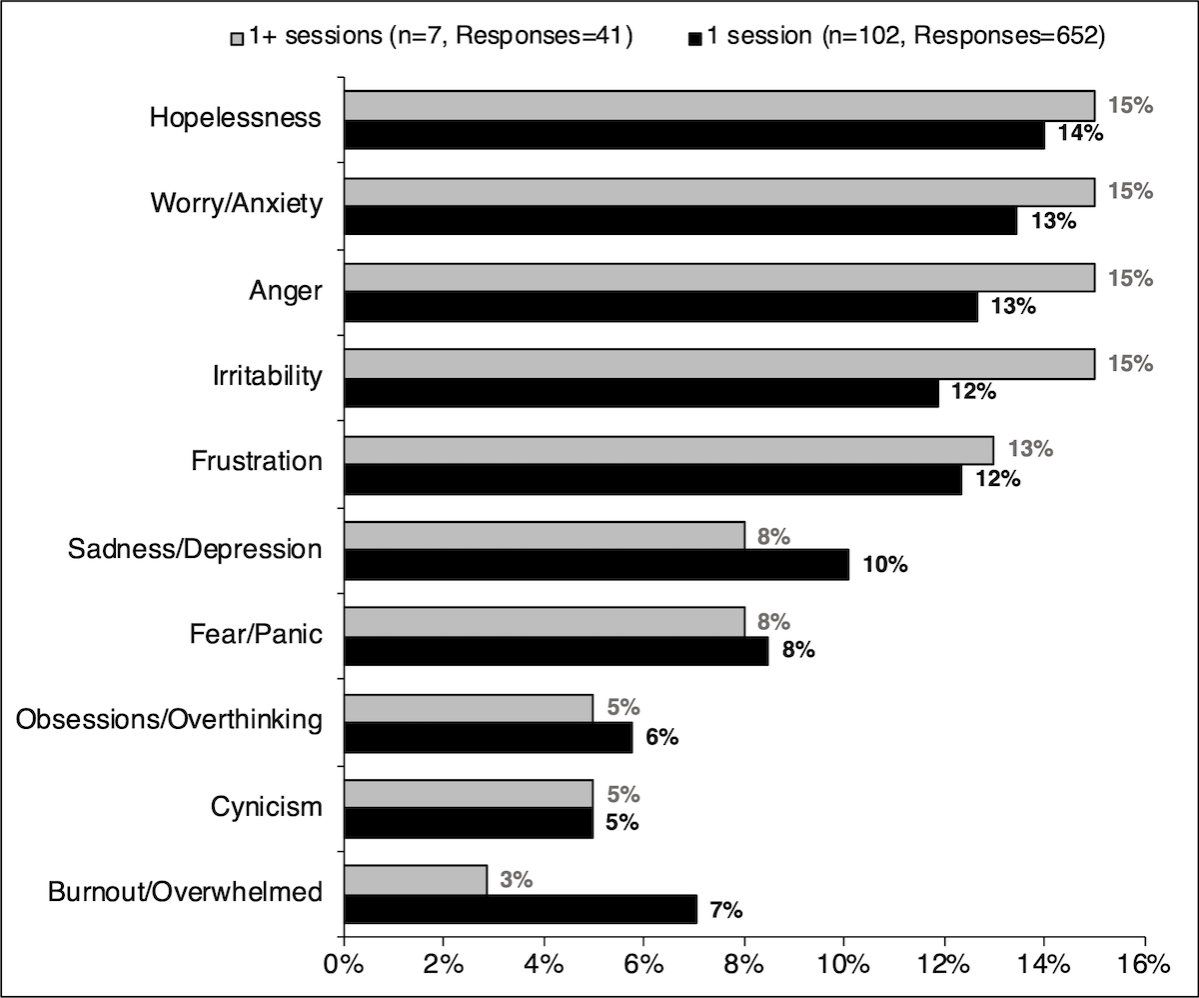
Most common emotional responses to perceived stressors by those who completed one versus multiple sessions.

The emotional response to perceived stressors were also grouped into 10 major categories: (1) hopelessness, (2) worry or anxiety, (3) anger, (4) irritability, (5) frustration, (6) sadness or depression, (7) fear or panic, (8) obsessions or overthinking, (9) cynicism, and (10) burnout or overwhelmed. The responses recorded allowed for multiple responses, and each response was placed into the appropriate category (ie, repeated instances within a category were counted individually). The most common responses were hopelessness, worry or anxiety, anger, irritability, and frustration among participants. The response breakdown can be found in Figure 4.

### Mood Module

#### Retention Rate and Session Duration

The time spent on each part of session one in the mood module was calculated in addition to the retention rate throughout the session. It had five major steps, including completing the PHQ-9, defining the problem, selecting a goal, brainstorming solutions, and developing an action plan. The time elapsed began when the participant started each section until the section was completed, including participant idle time and time away from the computer. The calculation included both individuals who completed the section in one and multiple sittings. For this reason, participant completion times totaling over 100 hours were not included in the calculation, and the mode and range are included in Table 2. Each section had a retention rate calculated based on the number of individuals who completed the previous section but did not complete the subsequent one (eg, percentage of those who completed “Possible solutions” but did not continue on to the “Action Plan”). The mode for the first four sections ranged from 7 minutes to 15 minutes, the highest retention rate occurred between the possible solutions and action plan, and the highest attrition rate occurred between the PHQ-9 completion and problem definition.

**Table 2:** Mood session one duration and attrition rate.

Item	PHQ-9^a^ completion (n=289)	Problem definition (n=126)	Goal selection (n=87)	Possible solutions (n=56)	Action plan (n=42)
Time spent (h:min), mean (range)	1:22 (0:03-72:25)	0:34 (0:04-18:41)	0:37 (0:03-72:25)	2:28 (0:06-47:36)	1:07 (0:12-3:23)
Mode (min)	0:07	0:09	0:10	0:15	N/A^b^
Retention rate (%)	Baseline	43.6	69.0	64.4	75.0

^a^PHQ-9: Patient Health Questionnaire-9.

^b^N/A: not applicable.

#### Problems Selected

**Figure 5:**
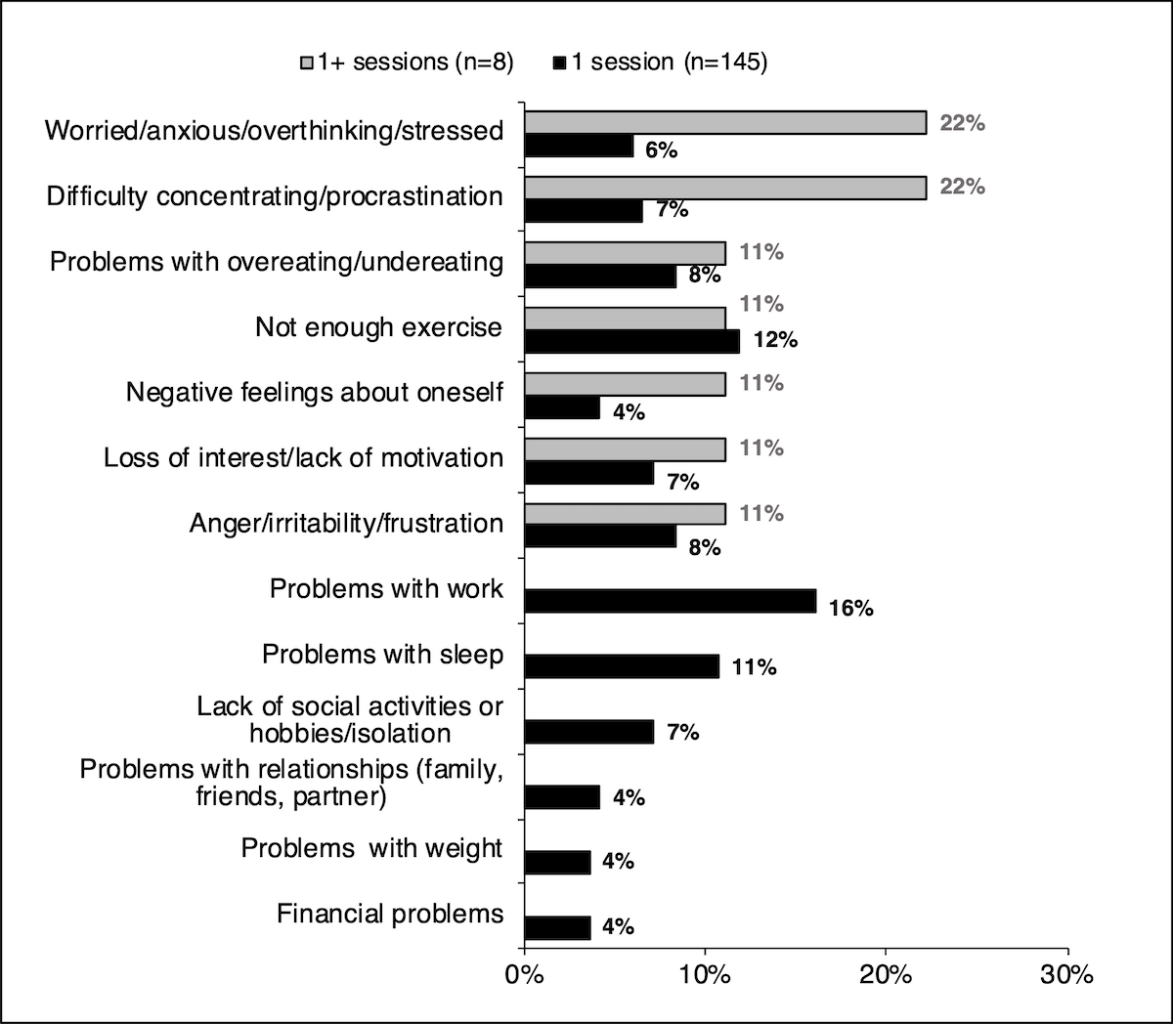
Most common problems selected by participants in the mood module.

The type of problems selected by participants in the program was evaluated to better understand the kind of issues experienced by participants and differences, if any, among individuals who only completed one session (n=145) versus those who completed multiple sessions (n=8). The problems were grouped into 13 categories: (1) worried, anxious, overthinking, or stressed; (2) difficulty concentrating or procrastination; (3) problems with overeating or undereating; (4) not enough exercise; (5) negative feelings about oneself; (6) loss of interest or lack of motivation; (7) anger, irritability, or frustration; (8) problems with work; (9) problems with sleep; (10) lack of social activities or hobbies, or isolation; (11) problems with relationships (family, friends, partner); (12) problems with weight; and (13) financial problems. Those who completed multiple sessions ranked worried, anxious, overthinking, or stressed and difficulty concentrating or procrastination as the highest among the major problems. In contrast, those who completed just one session had problems with work and not enough exercise as the highest ranked. The complete analysis is shown in Figure 5.

### Effects on Depression

**Figure 6:**
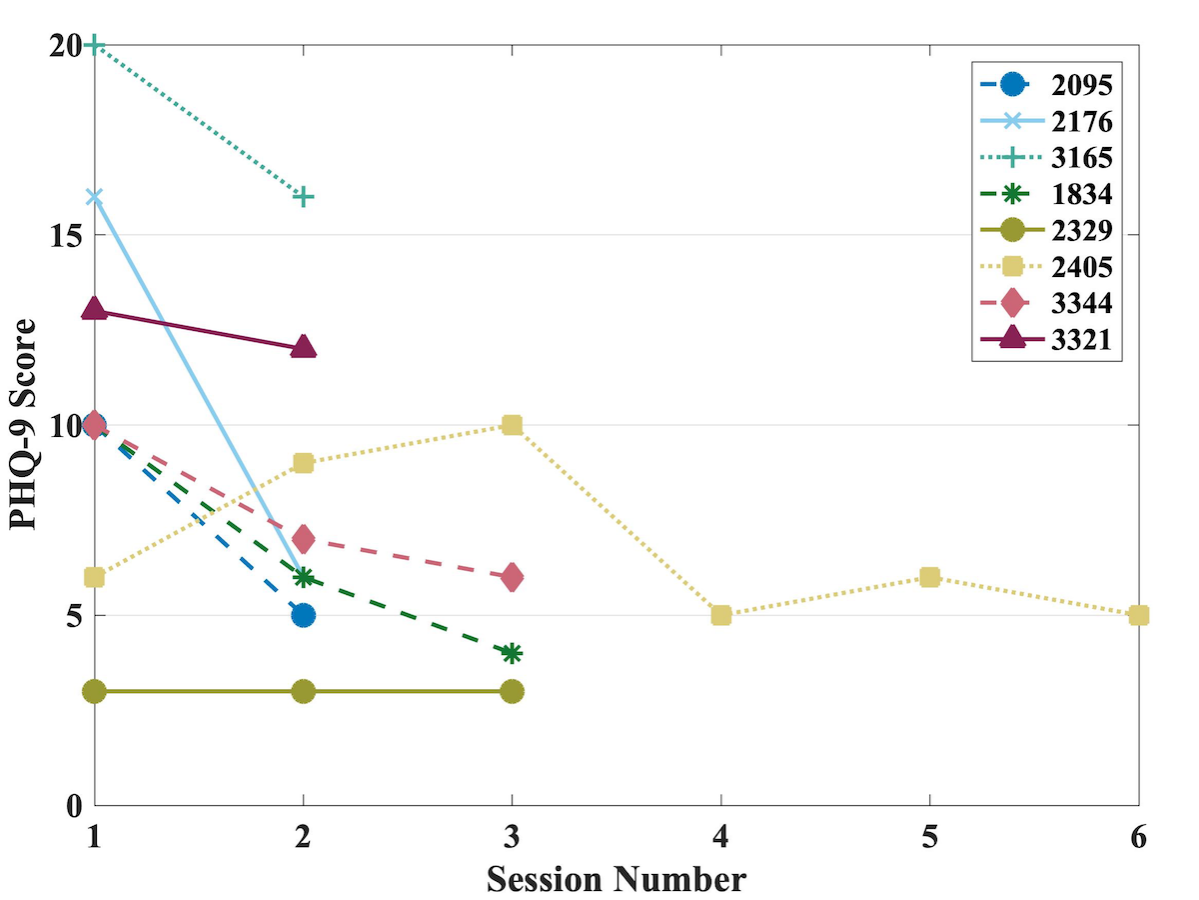
Change in depression during sessions one through six measured with PHQ-9 scores. The figure legend's four-digit values correspond to the identification numbers randomly assigned to users during program registration. PHQ-9: Patient Health Questionaire-9.

The change in depression severity levels was analyzed using the PHQ-9. The mean PHQ-9 score baseline for session one, including all participants (n=289), was 9.98 (SD 6.23). The average PHQ-9 of session one for those who returned for more than one session (n=8) was 11.0 (SD 5.4) while session two (n=8) was 8.0 (SD 4.2). The difference in scores between the first two sessions, analyzed using the Wilcoxon signed rank test, was not significant (*P=*.06). The depression severity levels between the two sessions decreased by an average of 20.0% (SD 35.2%). The mean PHQ-9 score for session three (n=4) was 5.8 (SD 3.1), session 4 (n=1) was 5.0, session 5 (n=1) was 6.0, and session 6 (n=1) was 5.0. The individual analysis of the PHQ-9 scores across sections one through six is shown in Figure 6.

The linear mixed-effects model analysis of the relationship between time and PHQ-9 scores with time as the fixed effect and the intercept of subjects as random effects showed depressive symptoms decreased significantly over time (β_1_=–.15, SE 0.056, 95% CI –0.27 to –0.038; *P*=.01).

#### Enjoyable Activities Selected

At the end of each session, participants were asked to schedule enjoyable activities to be completed until the next session. The activities were classified based on the different types of joy-related feelings associated with different activity categories [35]. The categories included social (activities involving interaction with others), intellectual (school-related activities, going to a museum, or going to a concert), basic needs (activities that provide essential elements that their body requires, such as eating or bathing), physical (any activity that promotes physically active movements of the body), nurturance (activities involving emotional or physical care of others), mastery (activities that involve learning or improving one’s skills), spirituality (religion-related activities or other forms of connection to the divine), and entertainment (miscellaneous activities such as watching TV or going to places not covered by the aforementioned categories) [35].

**Figure 7:**
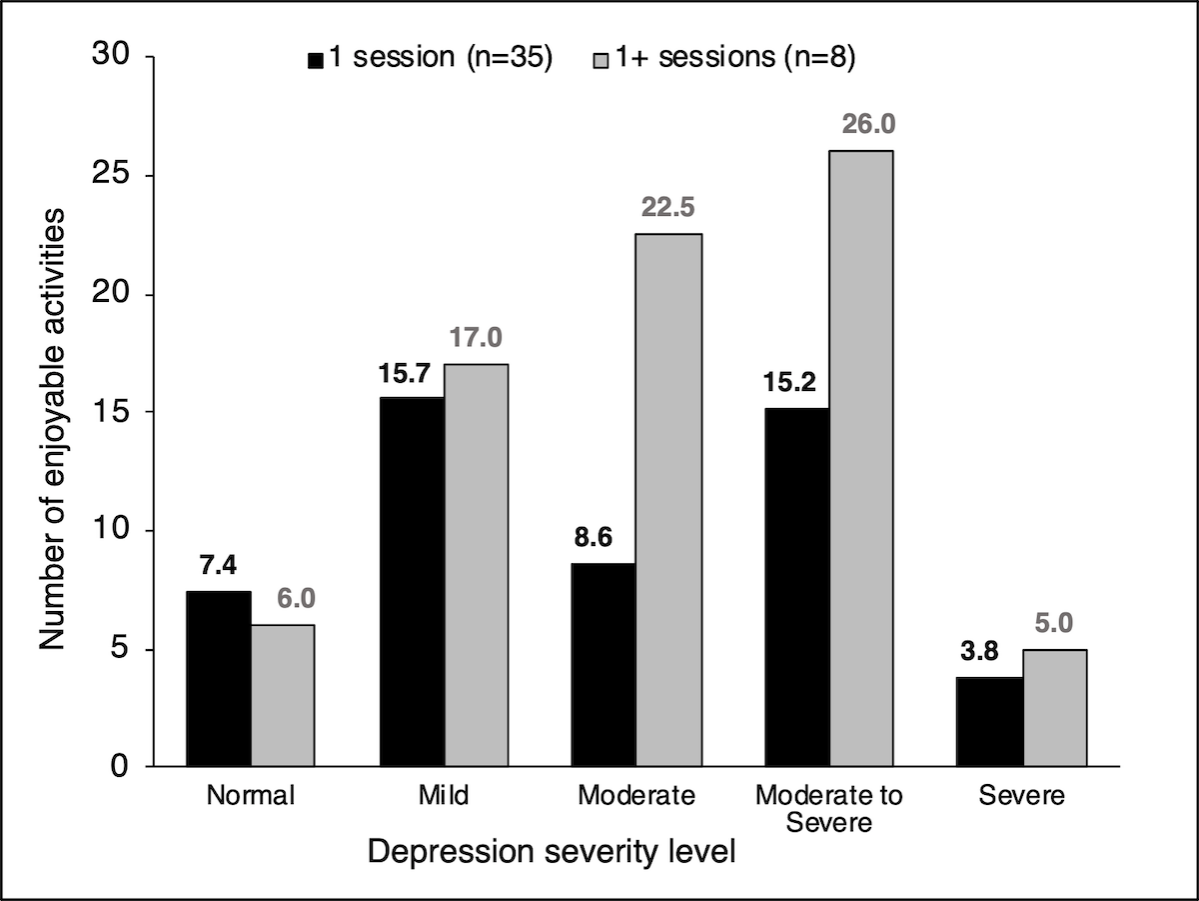
Mean number of activities selected by those who completed one versus multiple sessions based on Patient Health Questionaire-9 depression levels.

The average number of enjoyable activities selected by participants was analyzed based on the PHQ-9 depression level scores and the number of sessions completed. Those with higher depression levels who completed more than one session (n=8) selected more activities on average than those with lower PHQ-9 scores who completed only one session (n=35). A visual analysis is shown in Figure 7.

**Figure 8:**
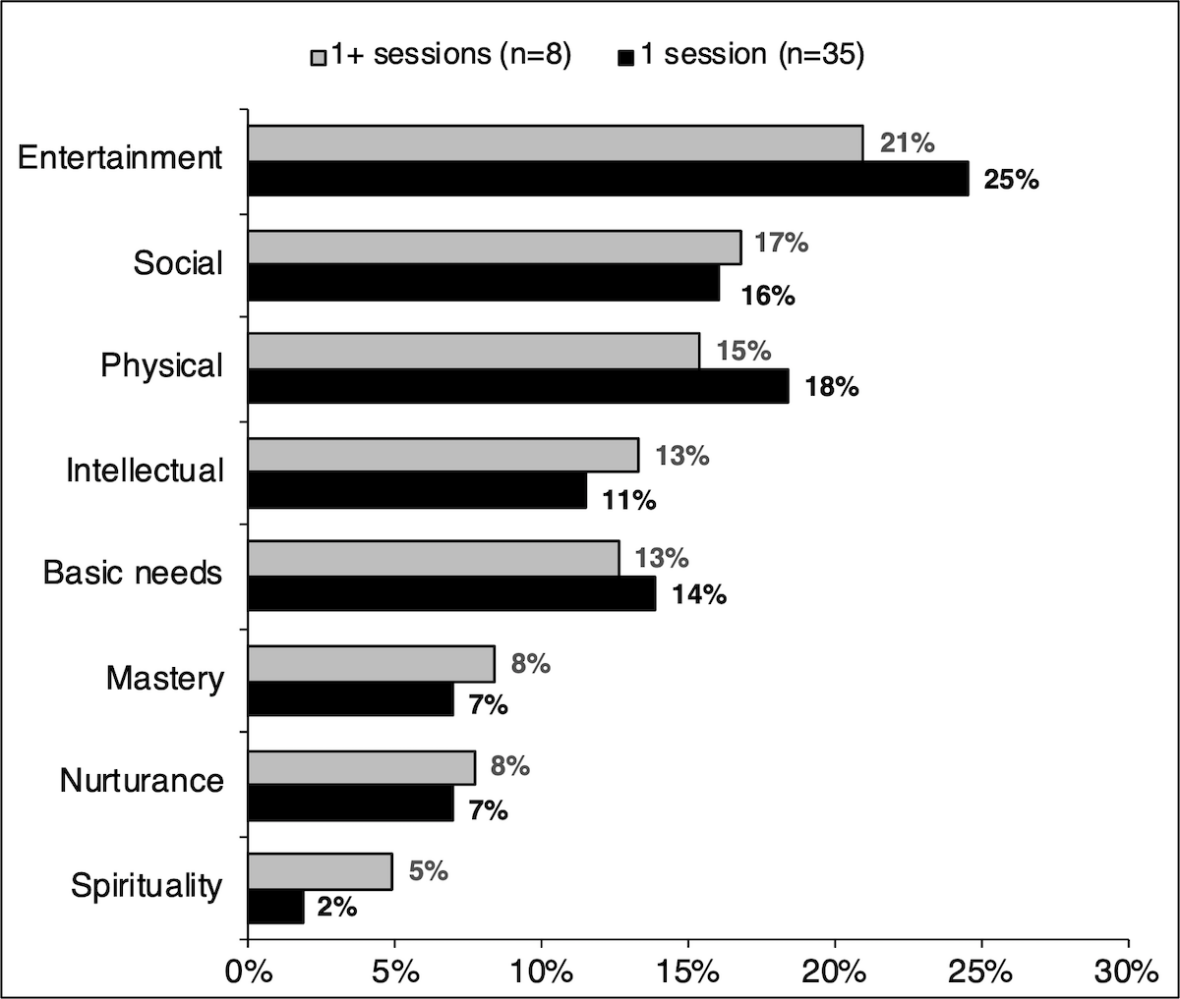
Types of enjoyable activities selected by those who completed one versus multiple sessions.

The most common type of activities selected among participants were entertainment, social, and physical activities. The response breakdown for each category is shown in Figure 8.

### Usability and Acceptability

This secondary analysis consisted of evaluating the usability and acceptability of the program and differences perceived by those who completed session one and did not return to the program versus those who came back to complete more than one session.

Lower PSSUQ scores indicated a more agreeable usability experience. A total of 22 individuals completed this questionnaire at the end of session one in the mood module, of which 5 individuals went on to complete more than one session. The mean overall usability score after session one given by those who completed one session (n=17) versus those who completed multiple sessions (n=5) was 2.7 (SD 1.8) and 3.2 (SD 2.2), respectively.

A total of 10 individuals completed the usability questionnaire at the end of session one in the stress module, of which 4 of those participants completed more than one session. The mean overall score after session one given by those who completed one session (n=6) and multiple sessions (n=4) was 3.4 (SD 2.5) and 4.1 (SD 2.4), respectively. Mean scores for each one of the PSSUQ subscales are presented in Table 3.

**Table 3:** PSSUQ scores for self-guided treatment of the mood (n=22) and stress (n=10) modules of individuals who completed one session versus those who completed multiple sessions.^a^

Items	Mood, mean (SD)					Stress, mean (SD)					PSSUQ^b^ norms, mean (99% confidence limits)
	1 session (n=17)	*P* value	>1 session (n=5)	*P* value	1 session (n=6)		*P* value	>1 session (n=4)	*P* value		
System use (Q1-Q8)	2.6 (1.9)	.004	2.9 (2.4)	.19	3.3 (2.5)		.34	3.9 (2.5)	.50	2.8 (2.6, 3.0)	
Information quality (Q9-Q15)	2.6 (1.9)	.004	3.4 (2.2)	.50	3.3 (2.5)		.34	4.2 (2.2)	.88	3.0 (2.8, 3.2)	
Interface quality (Q16-Q19)	3.0 (1.7)	.03	3.6 (2.2)	.50	3.7 (2.4)		.34	4.3 (2.5)	.88	2.5 (2.3, 2.7)	
Overall (Q1-Q19)	2.7 (1.8)	.004	3.2 (2.2)	.19	3.4 (2.5)		.34	4.1 (2.4)	.88	2.8 (2.6, 3.0)	

^a^Items are scored on a 7-point scale ranging from “Strongly agree” (1) to “Strongly disagree” (7), so lower scores are better. A sign test was performed to evaluate whether the median acceptability scores were significantly lower than a median of 4 (neutral).

^b^PSSUQ: Post-Study System Usability Questionnaire.

Higher scores on the AST questionnaire indicate a higher degree of acceptability. A total of 25 individuals completed the questionnaire at the end of session one in the mood module. The mean acceptability score given by those who completed session one only (n=20) was 5.7 (SD 0.7), and the mean score given by individuals who completed multiple sessions (n=5) was 5.9 (SD 0.4). Both of these were significantly different from neutral.

A total of 12 individuals completed the questionnaire at the end of session one in the stress module, of which 5 individuals completed multiple sessions. The mean acceptability score for the stress module after session one given by those who completed one session (n=7) was 5.5 (SD 0.7), and the mean score given by those who completed multiple sessions (n=5) was 5.3 (SD 0.4). These scores were significantly different from neutral.

The mean acceptability score for the conflict module (n=20) was 5.3 (SD 1.2) and statistically significant from neutral. Mean scores for each AST item are presented in Table 4.

**Table 4:** Acceptability of Self-Guided Treatment Questionnaire scores for the mood, stress, and conflict modules.^a^

Acceptability item	Mood, mean (SD)					Stress, mean (SD)					Conflict (n=20), mean (SD)		*P* value
	1 session (n=20)	*P* value	>1 session (n=5)	*P* value	1 session (n=7)		*P* value	>1 session (n=5)	*P* value				
I felt comfortable using the computer.	6.6 (0.8)	<.001	6.8 (0.4)	.03	6.1 (1.1)		.02	6.3 (1.0)	.06	5.7 (1.6)		<.001	
Doing [problem-solving treatment/stress management/conflict training] using this program was acceptable to me.	6.2 (0.8)	<.001	6.2 (0.4)	.03	6.1 (1.1)		.02	6.2 (0.8)	.03	5.7 (1.4)		<.001	
Using the program helped me to do [problem-solving treatment/stress management/conflict management].^b^	6.1 (0.9)	<.001	6.4 (0.5)	.03	5.6 (1.1)		.03	6.2 (0.8)	.03	5.6 (1.5)		.01	
I would rather do [problem-solving treatment/stress management/conflict management] with a therapist than with the computer.^c^	4.5 (1.6)	.09	4.0 (1.4)	.75	4.6 (1.6)		.31	4.6 (0.5)	.13	4.6 (1.6)		.03	
I would rather use a computer to help myself privately than go to a therapist.	4.3 (1.5)	.21	5.0 (1.2)	.13	4.9 (1.1)		.13	4.2 (0.8)	.50	4.8 (1.5)		.046	
Computer programs can help people with emotional problems such as depression.^d^	5.6 (1.1)	<.001	5.6 (1.0)	.06	5.4 (1.0)		.03	4.6 (0.5)	.13	5.4 (1.5)		<.001	
I would feel comfortable using this program without a clinician’s supervision.	5.6 (1.4)	.002	5.6 (1.5)	.19	6.0 (1.0)		.02	5.6 (1.1)	.06	N/A^e^		N/A	
I felt safe using the program to do problem-solving treatment.^f^	6.2 (0.9)	<.001	6.6 (0.5)	.03	6.0 (1.0)		.02	5.8 (0.8)	.03	N/A		N/A	
I would feel safe doing self-guided treatment for [depression/stress] on my own without a clinician’s supervision.	5.6 (1.2)	<.001	5.0 (1.2)	.13	5.6 (1.1)		.03	5.2 (1.3)	.13	N/A		N/A	
I would recommend this program to a friend [who was also in need of treatment for depression].^g^	5.9 (1.0)	<.001	5.6 (0.9)	.06	4.7 (1.8)		.11	4.4 (0.5)	.25	5.7 (1.4)		<.001	
Using a program like this could help someone to feel better.^h^	6.0 (0.8)	<.001	6.4 (0.5)	.03	5.4 (1.0)		.03	5.2 (0.8)	.06	5.8 (1.4)		<.001	
I believe I would feel comfortable using the program at home on my own computer.	6.3 (0.9)	<.001	6.6 (0.5)	.03	6.1 (1.0)		.02	6.0 (1.0)	.03	5.8 (1.6)		<.001	
I felt comfortable answering questions about [depression/stress] symptoms using this program.	6.2 (0.9)	<.001	6.6 (0.5)	.03	5.9 (0.9)		.02	5.6 (1.1)	.06	N/A		N/A	
I would feel comfortable using this program without a clinician’s assistance.	5.8 (1.4)	<.001	5.8 (1.6)	.19	6.0 (1.0)		.02	5.6 (1.1)	.06	N/A		N/A	
Overall score	5.7 (0.7)	<.001	5.9 (0.4)	.03	5.5 (0.7)		.03	5.3 (0.4)	.03	5.3 (1.2)		<.001	

^a^The conflict module contains 9 items. Items are scored on a 7-point scale ranging from “Strongly disagree” (1) to “Strongly agree” (7), so higher scores are better. A sign test was performed to evaluate whether the median acceptability scores were significantly higher than a median of 4 (neutral).

^b^Question slightly modified in conflict module to “Using the program helped me improve my conflict management skills.”

^c^Question slightly modified in conflict module to “I would rather do conflict-management training with a person rather than with a computer.” Score is inverted when calculating final overall score.

^d^Question slightly modified in conflict module to “Computer programs can help people improve their conflict management skills.”

^e^N/A: not applicable.

^f^The problem-solving treatment in the stress module focused on stress management and resilience training, while problem solving in the mood module focused on simulated live cognitive behavioral therapy treatment.

^g^Question slightly modified in conflict module to “I would recommend this program to a friend.”

^h^Question slightly modified in conflict module to “Using a program like this could help someone improve their conflict management skills.”

The difference in response patterns was further evaluated between individuals who completed the first session only and those who completed multiple sessions for the item “I would rather use a computer to help myself privately than go to a therapist.” In the stress module, 43% (3/7) of individuals who completed only one session chose “agree,” while 57% (4/7) chose “neither agree nor disagree.” A third of participants who completed multiple sessions chose “somewhat agree,” 50% (3/6) chose “neither agree nor disagree,” while 17% (1/6) chose “somewhat disagree.” In the mood module, 45% (9/20) of participants who completed only the first session agreed with the statement and 30% (6/20) were neutral, while 25% (5/20) disagreed with the item. Individuals who completed multiple sessions in the mood module mostly agreed with the item at a rate of 60% (3/5), while 40% (2/5) remained neutral. The complete analysis can be found in Table 5.

**Table 5:** Analysis of responses to the Acceptability of Self-Guided Treatment item “I would rather use a computer to help myself privately than go to a therapist.”

Item response	Stress 1 session (n=7), n (%)	Stress >1 session (n=6), n (%)	Mood 1 session (n=20), n (%)	Mood >1 session (n=5), n (%)	Conflict (n=20), n (%)
Strongly agree	0 (0)	0 (0)	1 (5)	1 (20)	2 (10)
Agree	3 (43)	0 (0)	3 (15)	0 (0)	4 (20)
Somewhat agree	0 (0)	2 (33)	5 (25)	2 (40)	4 (20)
Neither agree nor disagree	4 (57)	3 (50)	6 (30)	2 (40)	7 (35)
Somewhat disagree	0 (0)	1 (17)	1 (5)	0 (0)	3 (15)
Disagree	0 (0)	0 (0)	4 (20)	0 (0)	0 (0)
Strongly disagree	0 (0)	0 (0)	0 (0)	0 (0)	0 (0)

## Discussion

### Principal Findings

This study evaluated the use and effectiveness patterns of a free and unrestricted web-based program designed to provide cognitive behavioral–based approaches to stress, depression, and conflict management during the COVID-19 pandemic. The program provided insight into the kinds of problems people were experiencing during the pandemic and seeking help for. In the stress module, the greatest source of stress was conflicts with family or spouse. Feelings of hopelessness, worry, anxiety, and anger were common. The problems selected for the mood program focused on issues of worry, anxiety, overthinking, and stress. Those who returned to complete more than one mood session tended to have problems with worry, stress, and anxiety.

Those who returned to complete more than one stress or mood module showed significant reductions in self-reported stress and depression, although they did not rate the program as high for usability as those who completed only one session. Individuals who completed multiple sessions of the mood module also rated the program higher for acceptability than those who completed only the first session and tended to agree that they would rather help themselves privately with a computer rather than go to a therapist. For the stress module, individuals who completed only one session rated the program with slightly higher acceptability scores than those who returned to complete multiple subsequent sessions. On average, users thought the conflict training was useful and would recommend it to a friend. In the conflict module, the interest-based negotiation exercise was the highest rated.

The usability score describes the perceived ease of use of the technology applied in this program. It is based on a scale of 1 to 7 (ranging from “strongly agree” to “strongly disagree”), where a higher usability score infers a lower perceived ease of use by the participant. A psychometric evaluation of the PSSUQ established norms for this questionnaire [36]. The amount of usability data collected on the program was limited because the usability questionnaire was moved to after the first stress or mood session late in the study. The largest set of usability data was for session one of the mood program (n=17), which showed that the mood program was rated as significantly better than the norm (lower than the lower limit of the 99% confidence limit) for information quality and was within norms for system use and overall usability. The program, however, was rated worse than the norm on interface quality, although still significantly better than neutral (4) on the scale. The usability data on the stress module and for those who completed more than one visit was limited by low participant numbers (4-6 participants). There was a tendency, however, for the stress program to be rated lower on usability than the mood program. This indicates that further usability assessments would be worthwhile to identify the areas needing improvement.

The PSSUQ score results provided one surprising finding. Those who completed more sessions (stress: n=4; mood n=5) perceived the program as less usable than those who completed just one session (stress n=6; mood n=17). This result was unforeseen, as one would expect participants rating the programs with a low usability score would be likely not to continue. The significance of this finding is limited by the small number of people in these groups who have usability data. Nevertheless, it is interesting that those who chose to continue were not necessarily those who rated the program highly for usability. For the mood module, one possible explanation for continuing was the participants feelings about whether they wanted to use a computer rather than a therapist. Although 25% of those who completed only one mood session disagreed with the statement “I would rather use a computer to help myself privately than go to a therapist,” none of those who did more than one session did.

Ratings on the AST showed that participants found all three modules to be acceptable. The mean score (on a scale of 1 to 7: strongly disagree to strongly agree) for the mood, stress, and conflict modules were 5.7 (SD 0.7), 5.5 (SD 0.7), and 5.3 (SD 1.2), respectively, which were all significantly different than average. These results are similar to mean scores for the same modules when they were used in isolated and confined environments (eg, the HI-SEAS and the Canadian Forces Arctic Station Alert) [18]. The HI-SEAS program was a Mars analog habitat where a crew of 6 people lived in a Mars-like habitat for 8 to 12 months. The Canadian Forces Alert station is the northernmost permanently inhabited place on Earth, and crews there are relatively isolated. The HISEAS and Alert research missions had similar results, with mean AST values of 5.7 (SD 0.9), 5.5 (SD 0.7), and 5.2 (SD 0.9), respectively. Noticeably, the scores given in the mood, stress, and conflict modules to the item “Doing problem-solving treatment/stress management/conflict training using this program was acceptable to me” were 6.2 (SD 0.8), 6.1 (SD 1.1), and 5.7 (SD 1.4), respectively, on a 1 to 7 scale, and scores to the item “I would recommend this program to a friend” were 5.9 (SD 1.0), 4.7 (SD 1.8), and 5.7 (SD 1.4), respectively, on a 1 to 7 scale. These results confirm the acceptability of the program with a general population and match results from previous studies [7,18,25,27,37].

The conflict module consisted of four sections, each concluding with a short questionnaire. The Conflict Introduction and Conflict Simulation Questionnaires were found to be interesting and valuable (mean 3.2, SD 1.0 and mean 3.2, SD 0.8, respectively; *P<*.001 on a 0-4 scale). From the Hypothesis Testing Questionnaire, participants indicated that the program was somewhat easy to understand (mean 2.5, SD 1.1; *P*=.048), and the feedback somewhat helped them understand why responses were correct or incorrect (mean 2.5, SD 1.3; *P*=.06). Lastly, the conflict-based negotiation activity was found to be both enjoyable (mean 3.1, SD 0.9; *P<*.001) and highly valuable for learning about conflict management (mean 3.3, SD 0.7; *P<*.001).

Symptoms of stress as measured by PSS scores between sessions one and two did not significantly decrease (*P=*.20); however, the scores indicated a significant improvement over time (*P=*.03). There was no difference in the response pattern of the selected stressful triggers and perceived emotional response to stress between those who completed one session versus multiple sessions. The major stressful triggers selected by participants included conflict with family or spouse and work or workload. Noticeably, COVID-19 and isolation, political climate, and time away from friends and family were also mentioned as stressful triggers. Lastly, the most common emotional response to perceived stressors were hopelessness, worry or anxiety, anger, and irritability.

There was a difference in the type of problems selected by participants who completed only one session versus those who went on to complete multiple sessions in the mood module. Specifically, those who completed only one session ranked problems with work and not enough exercise, and those who completed multiple sessions ranked worried, anxious, overthinking, or stressed and difficulty concentrating or procrastination as the major problems. Comparably, participants from a previous preliminary uncontrolled trial ranked relationships and financial problems as the highest [27]. This finding suggests more research is needed to identify if there are problems that are particularly suitable for being addressed by the program. The high dropout rate and limited number of people who completed more than one session restricted our interpretation of this finding.

There was a mean 20.0% (SD 35.2%) decrease in depression severity level, as measured by PHQ-9 scores between sessions one and two, which was not statistically significant (*P=*.60). However, there was a significant decrease in the depressive symptoms over time (*P=*.01). Finally, the average number of enjoyable activities selected by participants indicated that those with higher depression levels and who completed multiple sessions selected more enjoyable activities on average than those with lower PHQ-9 scores that completed only one session.

The results overall highlighted an ongoing problem with the use of online behavioral health tools. The fact that a large number of people came to view the programs based on reading or seeing a news article about the program indicates there is a strong interest in self-help tools for behavioral problems. In addition, 45% (9/20) of participants who completed only one session and 60% (3/5) of those who completed multiple sessions of the mood program indicated that they either agreed or strongly agreed with the statement that “I would rather use a computer to help myself privately than go to a therapist,” indicating that a significant number of people want to use a computer-based or web-based approach for issues like depression. The acceptability and usability of the programs was good, and those who completed activities showed improvement in self-reported stress and depression. Despite all this, however, very few people returned to continue their work on problem solving or stress management after the first visit. One possible explanation for this is that, no matter how acceptable, usable, or effective a program may be, CBT requires time and effort to be successful. Just like other activities that provide benefits but require effort (eg, dieting or exercising), maintaining the motivation to complete the programs is difficult. Programs like these likely need to be used within a supportive environment with a human touch that can provide encouragement and ongoing support [38].

### Limitations

Data collected was uncontrolled since this was essentially a pragmatic trial during a time when there was heightened interest in the program. In contrast to previous studies of computer-based and online-based interventions evaluated in the setting of clinical trials, we analyzed anonymous data of a self-guided freely available program where users were self-referred.

This evaluation of the PATH program has many limitations, with the most significant likely being the high attrition rate, which is consistent with other research on self-help interventions [11,34,39-42].

Additionally, the data reported here are only from those who completed an activity in the program and then opted to complete a questionnaire, resulting in a selection bias. We do not have data from those who did not complete activities or who may have stopped because of difficulties with either usability or acceptability. The small sample size for those completing multiple sessions restricted our interpretations as to whether this program had a significant effect on reducing mental health symptoms. Given these limitations, any generalization from these results needs to be evaluated with caution. Nevertheless, the opportunity to evaluate a program like this during the COVID-19 pandemic was unique.

The programs were initially developed for use at NASA, and the conflict module included examples and situations based in the space program. The high acceptability and usability ratings for the conflict program indicate that these examples could be understood and applied to the public outside of NASA. Nevertheless, feedback from some participants who completed the conflict module included questions regarding the meaning of some of the words used, such as “EVA” (extravehicular activities). The vocabulary included in some of the scenarios could have created a barrier, hindering the full understanding of the module.

The sample was also not well characterized because, to maintain participant anonymity, only minimal data were collected about the users. This makes it difficult to gauge how confounding variables such as ethnicity, socioeconomic status, or education level would affect the results and attrition rate of this study. Additionally, the Dartmouth PATH program had a high exposure within the Dartmouth hospital and college campus, which could have contributed to a sample skewed toward well-educated individuals. These factors could affect the results and dropout rates. Those with more experience with computers may be more likely to continue with the program compared to those with lower education backgrounds and less technological capability [43]. Future research will need to explore the generalizability of these findings by including a more in-depth demographic questionnaire at participant registration and an evaluation of computer and internet accessibility.

### Conclusions

During the COVID-19 pandemic, those who came to the program seeking self-help for behavioral programs and engaged with the program rated conflicts with family or spouse as a major source of stress. Feelings of hopelessness, worry, anxiety, and anger were commonly reported. The problems selected for the mood program focused on worry, anxiety, overthinking, and stress. Despite the high attrition rate, this study shows that an open-access online behavioral program aiming to treat depression, stress, and conflict management can be effective and rated highly for usability and acceptability by users. This suggests the main issue is not content but instead finding the best implementation strategy. A significant proportion of users reported that they preferred to address behavioral health programs on their own using an online resource. Those who returned for additional visits tended to report more issues with worry, stress, and anxiety, and on average rated the programs less usable than those who completed just one session. For acceptability, there were differences in response patterns between the mood and stress module. Although those who returned for more visits in the mood module rated the program as more acceptable, the opposite trend was true in the stress module. The ultimate value of this program as a stand-alone resource will depend on understanding the reasons for the low completion rates and addressing them effectively. Strategies such as progress tracking, professional support, and weekly reminders may help increase users’ adherence to online programs [44]. Attrition rate is also likely to decrease by integrating online-based interventions to a minimally guided approach through a therapist or coach. The minimum amount to which guidance will result in a significant improvement in retention rate and treatment efficacy will be an important focus of future studies.

## Appendix

Multimedia Appendix 1 Video demonstration of the PATH program.

## Abbreviations

AST: Acceptability of Self-Guided Treatment
CBT: cognitive behavioral therapy
EVA: extravehicular activities
HI-SEAS: Hawaii Space Exploration Analog and Simulation
ICBT: internet-delivered cognitive behavioral therapy
NASA: National Aeronautics and Space Administration
PHQ-9: Patient Health Questionnaire-9
PSS: Perceived Stress Scale-14
PSSUQ: Post-Study System Usability Questionnaire
